# Audiologists’ perceived value of ototoxicity management and barriers to implementation for at-risk cancer patients in VA: the OtoMIC survey

**DOI:** 10.1007/s11764-022-01316-7

**Published:** 2023-02-02

**Authors:** Dawn Konrad-Martin, Rachel Polaski, J. Riley DeBacker, Sarah M. Theodoroff, Angela Garinis, Cecilia Lacey, Kirsten Johansson, Rosemarie Mannino, Trisha Milnes, Michelle Hungerford, Khaya D. Clark

**Affiliations:** 1 National Center for Rehabilitative Auditory Research, VA Portland Health Care System, 3710 SW US Veterans Hospital Road, P5-NCRAR, Portland, OR 97239 USA; 2 Department of Otolaryngology/Head and Neck Surgery, Oregon Health & Science University, Portland, OR USA; 3 Department of Hematology/Oncology, VA Portland Health Care System, Portland, OR USA; 4 Charlie Norwood VA Medical Center, Augusta, GA USA; 5 Department of Medical Informatics and Clinical Epidemiology, Oregon Health & Science University, Portland, OR USA

**Keywords:** Ototoxicity, Hearing loss, Drug-related side effects and adverse reactions, Survey, Drug monitoring, Professional practice gaps

## Abstract

**Purpose:**

Platinum-based chemotherapies used to treat many types of cancers are ototoxic. Ototoxicity management (OtoM) to mitigate the ototoxic outcomes of cancer survivors is recommended practice yet it is not a standard part of oncologic care. Although more than 10,000 patients each year are treated with platinum-based chemotherapies at the US Veterans Health Administration (VA), the current state of OtoM in VA is not well-defined. This study reports on a national survey of VA audiologists’ perceptions regarding OtoM in cancer patients.

**Methods:**

A 26-item online survey was administered to VA audiologists and service chiefs across the VA’s 18 regional systems of care. Descriptive statistics and deductive thematic analysis were used to analyze the data.

**Results:**

The 61 respondents included at least one from each VA region. All reported they felt some form of OtoM was necessary for at-risk cancer patients. A pre-treatment baseline, the ability to detect ototoxicity early, and management of ototoxic effects both during and after treatment were considered high value objectives of OtoM by respondents. Roughly half reported routinely providing these services for patients receiving cisplatin and carboplatin. Respondents disagreed regarding appropriate hearing testing schedules and how to co-manage OtoM responsibilities with oncology. They identified barriers to care that conformed to three themes: care and referral coordination with oncology, audiology workload, and lack of protocols.

**Conclusions:**

Although VA audiologists value providing OtoM for cancer patients, only about half perform OtoM for highly ototoxic treatment regimens. The OtoMIC survey provides clinician perspectives to benchmark and address OtoM care gaps.

**Implications for cancer survivors:**

Collaboration between oncology and audiology is needed to improve current OtoM processes, so that cancer survivors can have more control over their long term hearing health.

**Supplementary Information:**

The online version contains supplementary material available at 10.1007/s11764-022-01316-7.

## Introduction

Platinum-based chemotherapeutics, such as cisplatin and carboplatin, are highly effective at treating a variety of common cancers including those of the bladder, bone, cervix, colon, endometrium, esophagus, head and neck, lung, ovaries, testes, pancreas, rectum, and stomach [1]. Unfortunately, platinum-based drugs are also highly ototoxic [2–7]. According to the 2018 Ototoxicity Working Group of Pharmaceutical Interventions for Hearing Loss, ototoxicity is defined as “…damage to the inner ear, targeting cochlear and vestibular structures and sensory function, due to exposure to certain pharmaceuticals, chemicals, and/or ionizing radiation” [8]. In a recent meta-analysis of data from over 5077 patients receiving cisplatin and/or carboplatin, hearing loss occurred in 38–49% of patients pooled across age and tumor type [9]. Cisplatin, the most ototoxic of these drugs, causes some combination of ototoxic hearing loss (prevalence 50–80% [10, 11]), tinnitus (prevalence 11–40%; [10, 12]), and/or balance problems (prevalence 0% to 50%; [7]) in the majority of those who receive it. The impact of ototoxicity on patient quality of life (QoL) is of increasing concern as cancer survivors are living longer and the number of people diagnosed with cancer each year continues to grow [13]. A recent review by Pearson et al. [14] found that QoL was lower in cancer patients with documented hearing loss and/or tinnitus following cisplatin chemotherapy than in patients who did not report these symptoms or who received another course of care. The one study (of the six identified and reviewed) that found no impact of chemotherapy exposure on QoL and hearing outcomes evaluated only self-reported hearing status [15]. Notably, a recent study of 1410 patients with testicular cancer treated with cisplatin-based chemotherapy showed that patients consistently underestimated the degree of their treatment-related hearing loss compared with contemporaneous audiometric observations [16]. Falchook and colleagues [17] found that oncologists also tend to underestimate the rate and severity of their patient’s chemotherapy side effects. This incongruence is most pronounced for non-visible symptoms like tinnitus. In the sample of 43 head and neck cancer patients receiving cisplatin-based chemotherapy, the oncology providers identified only seven of their patients as having ototoxicity-related tinnitus (16%) and considered all but one case to be mild. In contrast, 31 of the 43 patients (72%) self-reported developing tinnitus that they rated as mild, moderate, or severe using the National Cancer Institute Common Terminology for Cancer Adverse Events Patient-Reported Outcomes (CTCAE-PRO) [17]. Cancer survivors’ QoL should be considered in relation to ototoxicity especially as it pertains to the well-established correlations observed in the general population between uncorrected hearing loss and depression, isolation, social withdrawal, and cognitive decline [18–22]. Hearing loss also needs to be considered in relation to the provision of effective oncologic care, as hearing loss in older adults is associated with reduced levels of health literacy and increased patient–clinician miscommunication [23, 24]. In addition to the burden hearing loss places on the patient, there is also an associated economic burden. For adults living in the USA, it has been estimated that each new onset of hearing loss, tinnitus, or vestibular effects has a socioeconomic cost of over $350,000 [25]. To mitigate these impacts and ensure rapid intervention when indicated, a hearing health management approach through an ototoxicity management (OtoM) program is warranted [26].

Ototoxicity monitoring guidelines were put in place by the American Speech-Language-Hearing Association (ASHA) nearly three decades ago [27]. As shown in Table 1, other medical associations and public health entities have subsequently advocated not just for ototoxicity symptom surveillance but for programmatic OtoM. These guidelines advocate for including education and counseling, and ototoxicity assessments that take place before, during, and after treatment, and for the use of these data to promote timely provision of aural rehabilitation and chemotherapy dosage adjustments as indicated [28–32]. Unfortunately, ototoxic medications are commonly administered without the involvement of a hearing health provider on the care team.

**Table 1:** Position statements and guidelines regarding ototoxicity management (OtoM)

Association and document name	Year	Specialty area	Helpful content
American Academy of Audiology (AAA) Position Statement and Clinical Practice Guidelines: Ototoxicity Monitoring http://www.audiology.org [28]	2009	Audiology	• Overview of ototoxic medications • Expands ASHA 2004 by providing an outline of vestibulotoxicity monitoring, greater focus on rehabilitation
American Speech-Language-Hearing Association (ASHA) Guidelines for the Audiologic Management of Individuals Receiving Cochleotoxic Drug Therapy http://asha.org [27]	1994	Audiology	• Suggested procedures for monitoring with a focus on auditory function surveillance • First comprehensive OtoM procedures guideline
American Academy of Otolaryngology-Head and Neck Surgery (AAO-HNS) Position Statement: Ototoxicity http://www.entnet.org [29]	2015	Otolaryngology	• Role of otolaryngologists in ototoxicity monitoring advocates considering treatment changes when ototoxic event identified
Commission for the Early Detection of Childhood Hearing Loss (CODEPEH) Ototoxicity in childhood: Recommendations of the CODEPEH (Commission for the Early Detection of Childhood Hearing Loss) for prevention and early diagnosis https://bibliotecafiapas.es/genero/documentos-codepeh/ [33]	2022	Multidisciplinary	• Comprehensive monitoring suggestions for pediatric patients receiving any ototoxic medication • Algorithmic approach includes aural rehabilitation treatment options
Health Professions Council of South Africa (HPCSA) Audiological Management of Patients on Treatment that Includes Ototoxic Medications https://hpcsa.co.za [31]	2019	Audiology	• Outline of ototoxicity and vestibular toxicity management programs • Ototoxic monitoring flow chart • Includes vestibular point-of-care screenings • Inter-professional team collaboration chart
International Late Effects of Childhood Cancer Guideline Harmonization Group Recommendations for Ototoxicity Surveillance for Childhood, Adolescent, And Young Adult Cancer Survivors: A Report From The International Late Effects Of Childhood Cancer Guideline Harmonization Group In Collaboration With The PanCare Consortium [34]	2019	Multidisciplinary	• First attempt to unify pediatric OtoM guidelines internationally • Includes input from Children’s Oncology Group (US), Dutch Childhood Oncology Group, and UK Children’s Cancer and Leukaemia Group
International Society of Paediatric Oncology (SIOP) Recommendations for Age-Appropriate Testing, Timing, and Frequency of Audiologic Monitoring During Childhood Cancer Treatment: An International Society of Paediatric Oncology Supportive Care Consensus Report https://siop-online.org/ [32]	2021	Multidisciplinary	• Expands on 2019 international guidelines with specific protocol recommendations
World Health Organization (WHO) World Report on Hearing https://www.who.int/publications/i/item/world-report-on-hearing [30]	2021	Multidisciplinary	• Epidemiological data from different world regions • Emphasis on screening targeted to different age groups, including ototoxicity monitoring

In 2021, Konrad-Martin and colleagues evaluated the premise that OtoM should be the recommended standard of care for patients receiving cisplatin-based chemotherapy, using a randomized control trial conducted in the VA. The trial compared the efficacy of OtoM provided as a dedicated program using automated protocols that enabled patients to screen their own hearing in the oncology infusion unit, against a referral system for “usual care” ototoxicity monitoring administered through the audiology clinic. Although the research team made referrals to audiology for each participant in the usual care group, only one patient completed a monitoring visit prior to each cisplatin dose, the protocol recommended by US audiology governing bodies for patients receiving cisplatin [27, 28]. In contrast, most (83%) participants in the automated screening group completed the recommended screening. The two groups saw similar incidence of hearing shifts and self-reported hearing handicap and no significant difference in the estimated 2-year survival risk. Similarly, there was no difference across groups in the number of patients who adopted recommended hearing loss intervention (46% vs. 50% in the automated versus usual care groups, respectively) or who received a dose modification based upon the findings of audiological monitoring (1 patient in each group). Notably, this study included research visits to collect and identify ototoxicity outcomes for participants in both arms for comparison, and these outcomes were shared with the patient and their oncology teams for ethical reasons. Participants being informed of their hearing loss coupled with the low cost of audiological services in VA [33], may explain why so many participants actively followed up with audiology to obtain hearing aid services. The striking difference in uptake of recommended monitoring across groups indicates that OtoM as currently recommended poses significant barriers to access for patients, at least in the context of this study of 46 patients receiving cisplatin at a VA hospital [34]. A similar lack of effective OtoM delivery was also observed in a recent chart review of 379 patients who received cisplatin at UC Davis Medical Center. Although 24% of patients had hearing complaints documented in their medical record, only 4.5% received an audiometric evaluation during cisplatin treatment [35].

Many interventions shown to be effective in research studies fail to be successfully implemented in real-world healthcare settings. Barriers to successful implementation exist at multiple levels of healthcare delivery that must be understood and considered [36]. The Consolidated Framework for Implementation Research (CFIR) is a useful conceptual framework to guide the assessment of diverse implementation contexts, to inform the design of health care interventions, and to identify barriers and facilitators associated with the implementation of health care interventions [37]. Due to the inherent complexity of health care contexts and health care interventions, the CFIR framework describes intersecting factors that guide implementation across disparate contexts [38]. According to CFIR, intervention context and the implementation process are factors that can affect an intervention’s success [36]. Investigating clinical service gaps also is vital to understanding how clinical practices can be adapted to overcome current barriers and allow recommended care to become standard of care for at-risk patients. Research and federal healthcare system guidance shows that successful implementation of evidence-based interventions that impact patient outcomes require a common understanding of the problem as well as the alignment of goals among the key partners including provider groups, hospital leadership, and patients [39, 40]. Thoughtful consideration of audiologists’ and oncologists’ views on ototoxicity and its management in patients receiving cancer treatment is, therefore, important for the successful implementation of an OtoM program.

This report documents the development of the “Ototoxicity Management Through Interdisciplinary Care (OtoMIC) Survey” (provided in Appendix A of the online supplementary materials) and its use to gain VA audiologists’ perspectives that can be used to improve OtoM practices and protocols in VA. The VA is the largest integrated healthcare system in the USA [41]. In 2018, the most recent year with complete VA Cancer Registry data prior to the COVID-19 pandemic, 10,421 patients were treated with platinum-based chemotherapies (cisplatin 2443, carboplatin 5621, oxaliplatin 2357). The VA provides audiology services as high-priority, direct access care because tinnitus and hearing loss are the number one and number three most prevalent service-connected disabilities, respectively [42]. However, the current state of OtoM in VA is not well-defined. We hypothesized that evidence of OtoM service gaps similar to those identified in the usual care arm of our clinical trial [34] would be found throughout the VA. We further expected to find that provider perspectives would yield important insights on barriers in specific VA contexts, and how to overcome them.

## Methods

Requests for participation by VA audiology service chiefs/leads and one other audiologist per department were emailed to 128 individuals across the VA’s 18 integrated service networks (VISNs). Follow-up emails to service chiefs/leads were sent to VISNs that lacked responses following the initial request for participation. Respondents were surveyed between November and December of 2020 using a VA-approved secure web-based application called Qualtrics, and the survey took approximately 15 min to complete. Sixty-one respondents completed survey questions, and 57/61 respondents provided their VISN or specific VA location (shown in Appendix B). Two additional respondents provided information on barriers to OtoM through emailed responses bringing the total N for the qualitative analysis to 63. These two respondents indicated they did not fill out the survey itself.

### Survey development

**Fig. 1:**
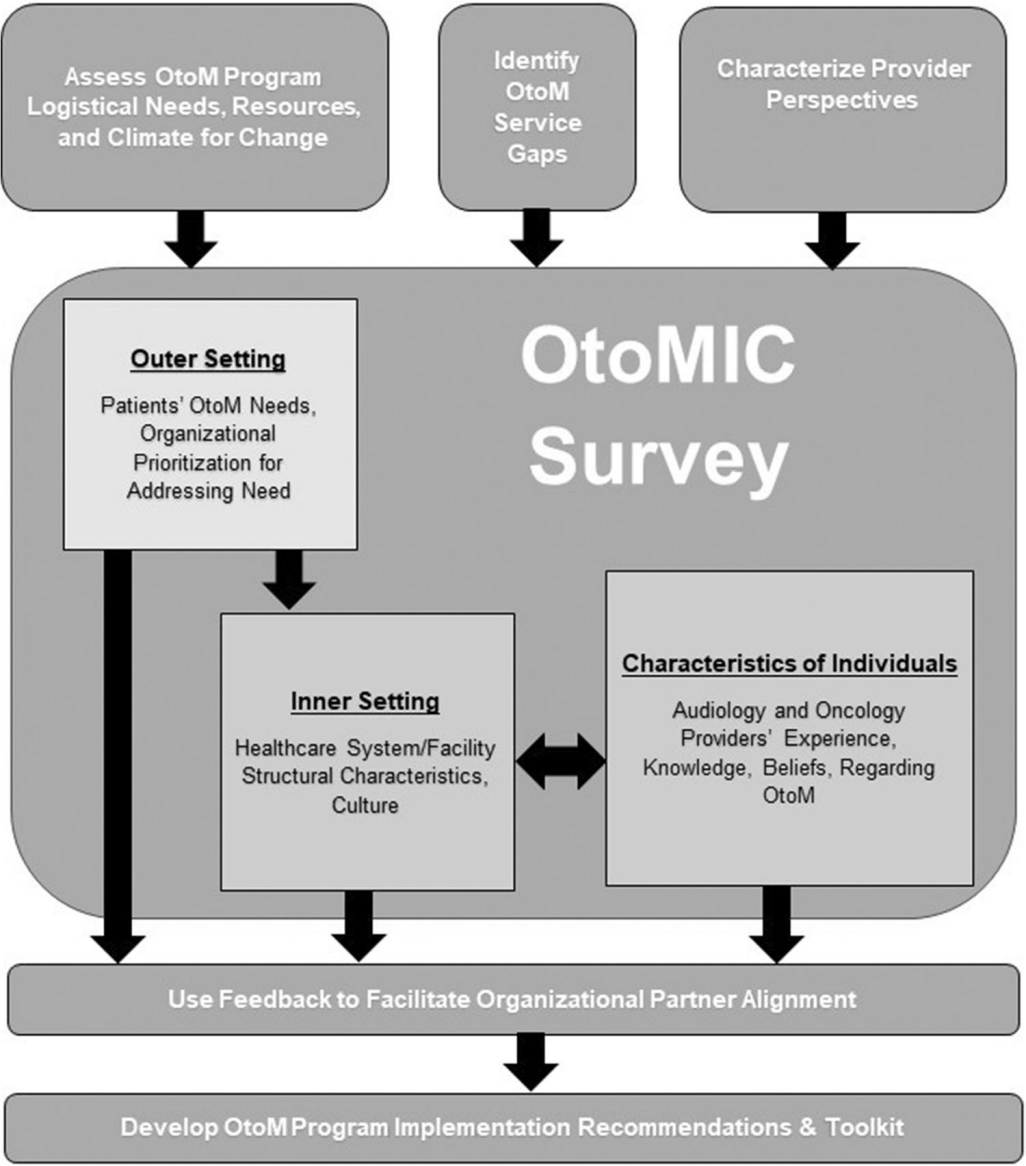
Objectives of the Ototoxicity Management Through Interdisciplinary Care (OtoMIC) survey. Flow chart shows relationships between the survey objectives, the CFIR constructs used to develop the survey, and the project goals

We developed a survey to inform OtoM implementation in multidisciplinary healthcare contexts. Survey objectives and questions were informed by three constructs from CFIR [36] (1) inner setting (e.g., structural characteristics of the healthcare system/facility), (2) outer setting (e.g., patients’ needs and resources committed by the organization to address these needs), and (3) individuals involved (e.g., provider experience, knowledge and beliefs, and other personal characteristics). The survey objectives were to identify: (a) demographic characteristics of the respondent (who could conceivably be any type of provider closely involved in the healthcare of patients receiving an ototoxic medical treatment); (b) number of patients receiving specific ototoxic medications; (c) knowledge of ototoxicity (e.g., prevalence, patient risk factors, symptoms); (d) current OtoM processes and protocols; (e) perspectives on the value and prioritization of OtoM; (f) OtoM implementation climate; and (g) barriers and facilitators associated with OtoM. Figure 1 is a schematic depicting the survey purpose and how it maps onto CFIR constructs in order to achieve our survey objectives and address our longer term goal of developing OtoM implementation recommendations and an associated toolkit specific for VA. The 26-item survey included multiple-choice, multiple-selection, rating-scale, ranking, and open-ended questions.

### Survey validation

The face validity of the survey was established by two different review panels. The first review panel consisted of a VA Audiology Chief, an Oregon Health & Science University Audiologist, a VA Oncology Chief, a VA Oncologist, and a VA Oncology Nurse Practitioner to ensure that the questions captured the salient dimensions of OtoM. This prompted revisions to the response options for a few of the multiple-choice questions, in addition to other minor edits. The second review was conducted by an Implementation Science/Health Service Delivery expert to assess question clarity and fit to the conceptual framework. The final version of the OtoMIC survey 1 (Appendix A) can be used or adapted for use with permission from the authors.

### Quantitative analysis

The quantitative data obtained from the survey were analyzed using descriptive statistics. Quantitative responses from the survey are reported as absolute frequency (number of respondents that selected each option), relative frequency (percentage of all responses to a question that included a given  response option), or the median response (non-parametric estimates of central tendency). All data were securely stored in compliance with VA policy.

### Qualitative analysis

A directed, deductive thematic analysis [43] was used to identify barriers and facilitators associated with OtoM. Two members of the project team (KC and RP) reviewed the data by reading and re-reading participant responses, which were downloaded directly from Qualtrics as a spreadsheet. During this immersive phase of reading the data, the analysts collaboratively developed a codebook to tag text related to OtoM barriers and facilitators. They then independently coded a subset of the responses using the codebook and met to compare any differences in coding. Next, to assess the reliability of our coding, the study PI (DK-M) reviewed the codebook, raw data, and coded output. Based on that review, two codes were collapsed into other codes, and the team determined that no further modifications were required. The lead analyst (KC) independently coded the remaining responses and connected the codes thematically, periodically checking in with the project team to discuss the meanings and revisions to the codebook. Atlas.ti software was used for all qualitative analyses.

## Results

### Demographics

Sixty-one respondents answered the demographic questions in the survey: VA name and geographic location, clinic type (VA Medical Center [VAMC], Community Based Outreach Center [CBOC]), and position (service chief/lead or staff member); of these, some chose not to provide specific location information. Forty-eight percent of the responses were from audiologists and the remaining 52% were from Speech Pathology and Audiology Service Chiefs, which corresponded with the expected response from one audiologist and one service chief or lead from each facility. Seventy-nine percent of respondents worked primarily at a VAMC only, and the other 21% worked at both a CBOC and a VAMC. Seventy-seven percent of the respondents had been practicing in their field for over 10 years and 15% of respondents had been practicing for 7–10 years. The geographical distribution of the 57 respondents who provided this information is shown in Appendix B. These 57 respondents included at least one from each of the VA’s 18 regional systems of care called Veterans’ Integrated Service Networks (VISNs). Four of the 61 respondents chose not to disclose their service location.

### Outer setting: patients’ OtoM needs

Questions related to the outer setting CFIR construct included those asking about Veterans OtoM needs and resources. Audiologists were asked approximately how many patients in their monthly caseload were receiving a platinum-based chemotherapy and/or radiotherapy. Appendix C shows the relative frequency distribution for this question. Over 70% of respondents reported that they saw fewer than 5 patients a month receiving cisplatin or carboplatin, and more than half also saw fewer than 5 patients a month receiving oxaliplatin or radiation.

Respondents were also asked what percentage of their patients experienced new or increased hearing loss, tinnitus, balance changes, or decreased quality of life because of an ototoxic agent. The reported presentation and impact of ototoxicity symptoms varied substantially. Despite a large range in responses, respondents indicated similar median incidence of new or increased tinnitus (20%), balance problems (17%), and hearing loss (18%) in their OtoM caseloads. Finally, the respondents reported that 29% of their OtoM patients experienced reduced quality of life due to the ototoxic impacts of their cancer treatment. These estimates can be seen in greater detail in Appendix D. When asked about their knowledge regarding the expected prevalence of ototoxicity, estimates for cisplatin were as low as 5%. On average, respondents indicated a lower ototoxicity symptom prevalence for cisplatin and carboplatin than reported in a large meta-analyses of treated patient cohorts [9].

### Inner setting: healthcare system/facility structural characteristics

The inner setting domain includes questions on the tasks that providers perform related to OtoM and the prioritization of ototoxicity monitoring. Table 2 shows a summary of what aspects of ototoxicity monitoring respondents routinely perform including baseline evaluation, monitoring hearing during treatment, and performing a follow-up exam after cessation of treatment. About half (39–64%) of respondents indicated “yes” to routinely performing all aspects of OtoM for cisplatin and carboplatin and “sometimes” or “no” for oxaliplatin and radiation. Responses to this question also displayed some differences between the responses of audiology chiefs/leads and the responses of their staff. Most audiology chiefs reported no implementation of key OtoM services for oxaliplatin and radiation, whereas audiologists’ responses varied more and indicated partial or full implementation for some services. Table 2 also shows respondent’s self-assessment regarding whether they routinely screen for tinnitus, and vestibular changes. As compared with hearing and tinnitus testing, balance testing was less often fully implemented.

**Table 2:** Level of implementation of OtoM clinical objectives. For each clinical objective, the possible responses, “Yes,” “Sometimes,” and “No,” were interpreted as “Fully implemented,” “Partially implemented” and “Not implemented” for the purposes of estimating the level of implementation for a given audiologist or audiology service. Responses were elicited by ototoxic exposure type. Numbers and percentages of respondents are tabulated separately for clinicians versus service chiefs/leads

Ototoxic agent	Fully implemented (yes)		Partially implemented (sometimes)		Not implemented (no)	
	Chief	Audiologist	Chief	Audiologist	Chief	Audiologist
Perform a baseline evaluation						
Cisplatin	9 (50%)	14 (56%)	8 (44%)	10 (40%)	1 (6%)	1 (4%)
Carboplatin	7 (44%)	11 (46%)	7 (44%)	11 (46%)	2 (13%)	2 (8%)
Oxaliplatin	3 (19%)	10 (42%)	4 (25%)	8 (33%)	9 (56%)	6 (25%)
Radiation	2 (12%)	6 (26%)	6 (35%)	10 (43%)	9 (53%)	7 (30%)
Monitor for hearing changes						
Cisplatin	12 (67%)	16 (62%)	5 (28%)	7 (27%)	1 (6%)	3 (12%)
Carboplatin	8 (47%)	13 (52%)	7 (41%)	9 (36%)	2 (12%)	3 (12%)
Oxaliplatin	1 (6%)	12 (48%)	6 (38%)	7 (28%)	9 (56%)	6 (24%)
Radiation	2 (12%)	11 (46%)	5 (29%)	6 (25%)	10 (59%)	7 (29%)
Perform a follow-up evaluation						
Cisplatin	9 (50%)	13 (52%)	8 (44%)	10 (40%)	1 (6%)	2 (8%)
Carboplatin	8 (47%)	10 (42%)	7 (41%)	11 (46%)	2 (12%)	3 (13%)
Oxaliplatin	2 (13%)	9 (38%)	5 (31%)	8 (33%)	9 (56%)	7 (29%)
Radiation	0 (0%)	8 (35%)	7 (44%)	9 (39%)	9 (56%)	6 (26%)
Screen for tinnitus						
Cisplatin	10 (56%)	16 (64%)	5 (28%)	5 (20%)	3 (17%)	4 (16%)
Carboplatin	8 (47%)	14 (58%)	5 (29%)	6 (25%)	4 (24%)	4 (17%)
Oxaliplatin	3 (19%)	11 (50%)	4 (25%)	6 (27%)	9 (56%)	5 (23%)
Radiation	2 (12%)	12 (52%)	6 (35%)	4 (17%)	9 (53%)	7 (30%)
Screen for vestibular changes						
Cisplatin	7 (39%)	12 (48%)	7 (39%)	5 (20%)	4 (22%)	8 (32%)
Carboplatin	6 (35%)	10 (42%)	6 (35%)	6 (25%)	5 (29%)	8 (33%)
Oxaliplatin	2 (13%)	8 (33%)	4 (27%)	6 (25%)	9 (60%)	10 (42%)
Radiation	2 (12%)	6 (26%)	6 (35%)	7 (30%)	9 (53%)	10 (43%)

**Fig. 2:**
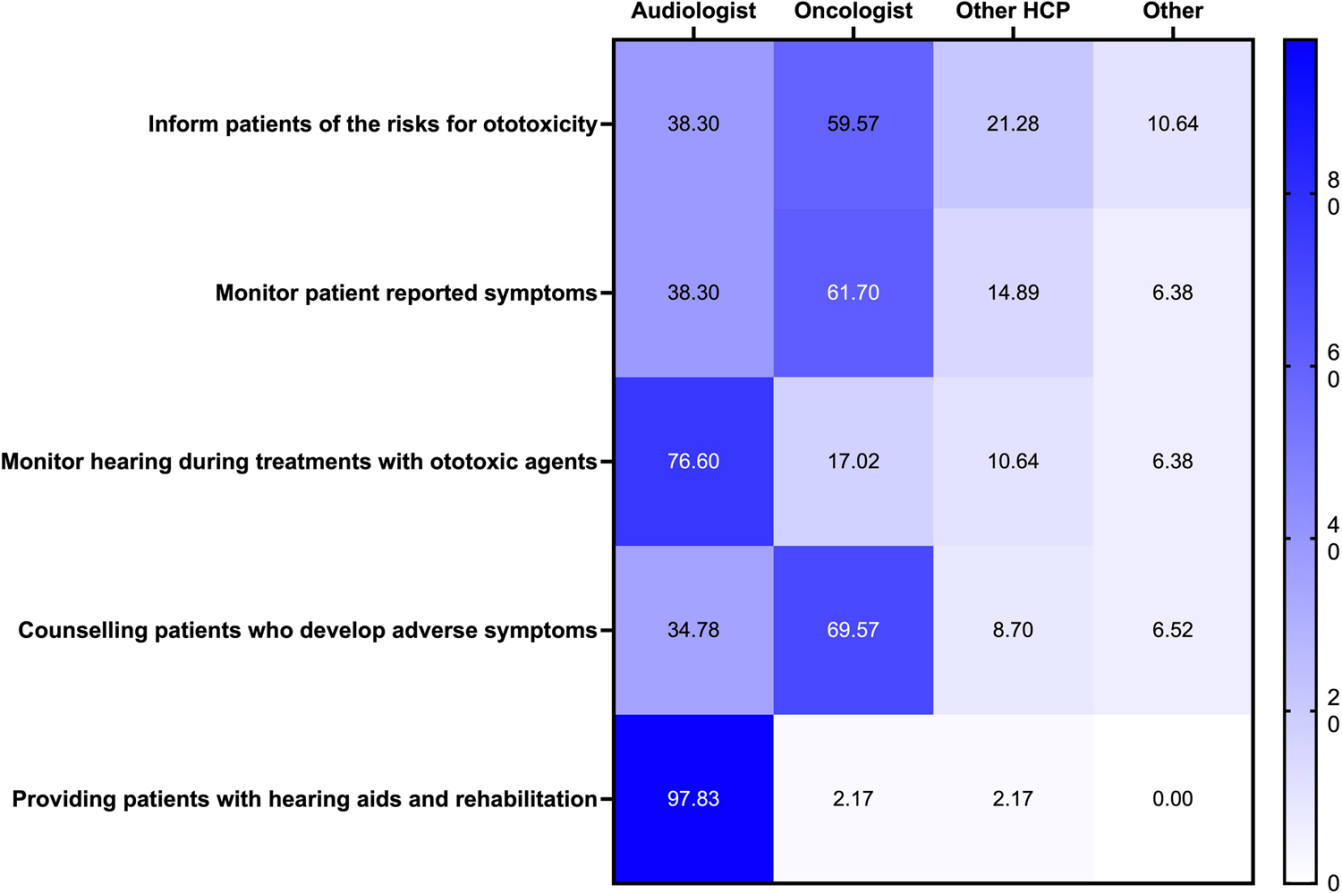
Multidisciplinary team member responsibilities for ototoxicity management (OtoM). Respondents were asked to select all applicable provider options (columns) for each OtoM objective (rows). Numeric and color scales show the percentage of respondents that indicated a given response option. HCP = Health Care Provider. *N = 46*. Also see Appendix E, which indicates audiologists’ perceptions regarding which objectives of OtoM are within their scope of practice

Several questions asked about team member responsibilities for aspects of OtoM. As shown in Fig. 2, the distribution of responses to question 17 reveals that many audiologists consider it part of their scope of practice to inform patients about the risks of ototoxicity, but only in a supporting role and look to oncologist to take the lead in these discussions. There was also a lack of alignment regarding who should communicate results of ototoxicity monitoring to the patient and be responsible for monitoring patient-reported ototoxicity symptoms. In a separate question about how patients access OtoM in a respondent’s facility, 89% of respondents indicated that the oncology team provides the referral (Appendix G).

### Individuals involved: providers’ experience, knowledge, and beliefs about OtoM

**Fig. 3:**
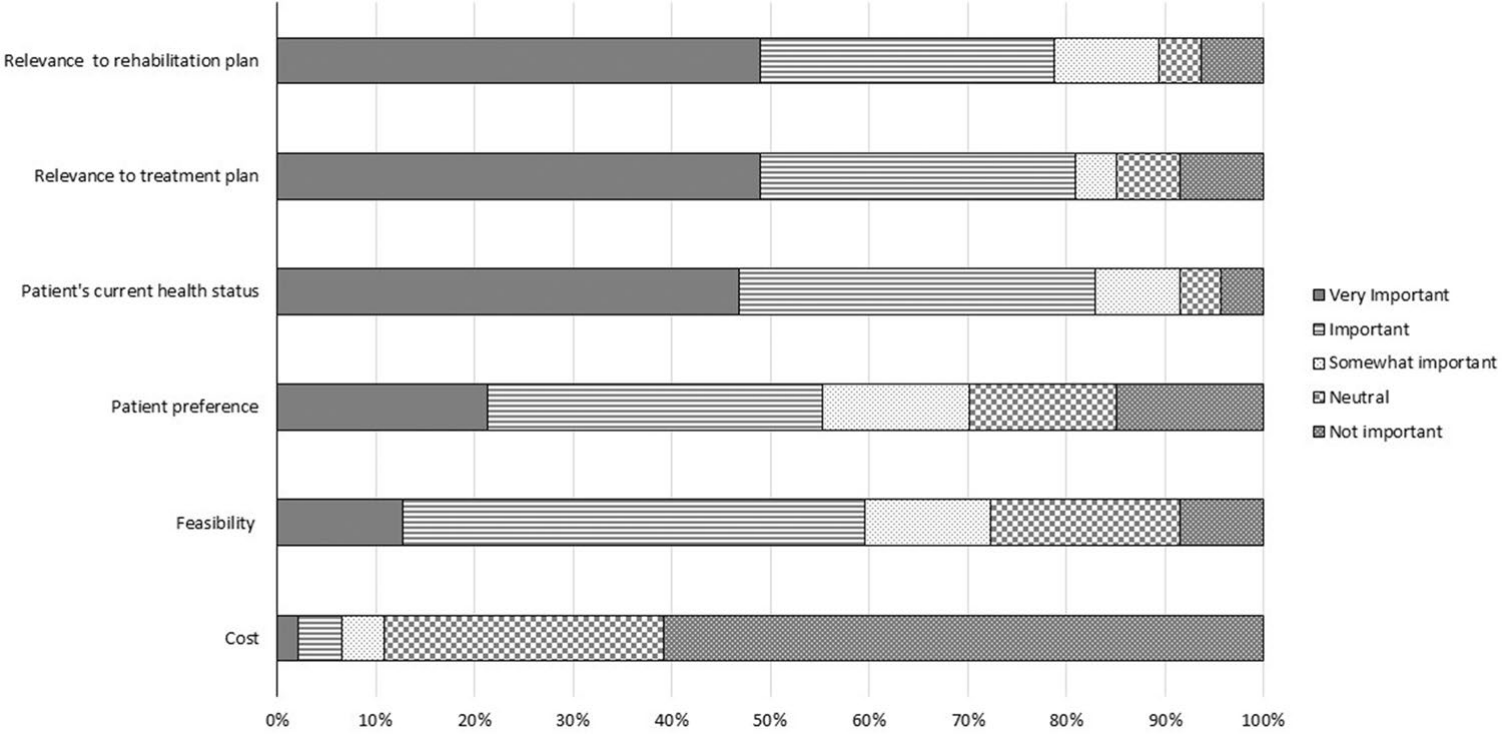
OtoM program considerations that should determine the schedule of ototoxicity monitoring tests. Figure is a relative stacked frequency plot in which the shading type indicates the level of importance of factors to consider (rows) when determining the schedule of ototoxicity symptom surveillance. *N = 46*

Survey respondents ranked hearing loss and balance dysfunction as the most and second most important non-lethal cancer treatment side effects to manage, respectively (Appendix F). These symptoms ranked higher than the ototoxic symptom of tinnitus and toxicities related to other body systems, though this may reflect a bias related to the survey audience. Respondents were asked to rate the importance of various factors for OtoM scheduling including cost, feasibility, patient health status, patient preference, and the ability of tests to impact rehabilitation or treatment. Responses are shown as stacked relative frequencies (percentages) in Fig. 3. The relevance of test results to rehabilitation and to the cancer treatment plan, and patient’s current health status were all rated as important or very important by about 80% of respondents. In contrast, cost was rated as not important or neutral by nearly 90% of respondents. Notably, audiologists rated feasibility as less important than all factors other than cost.

**Fig. 4:**
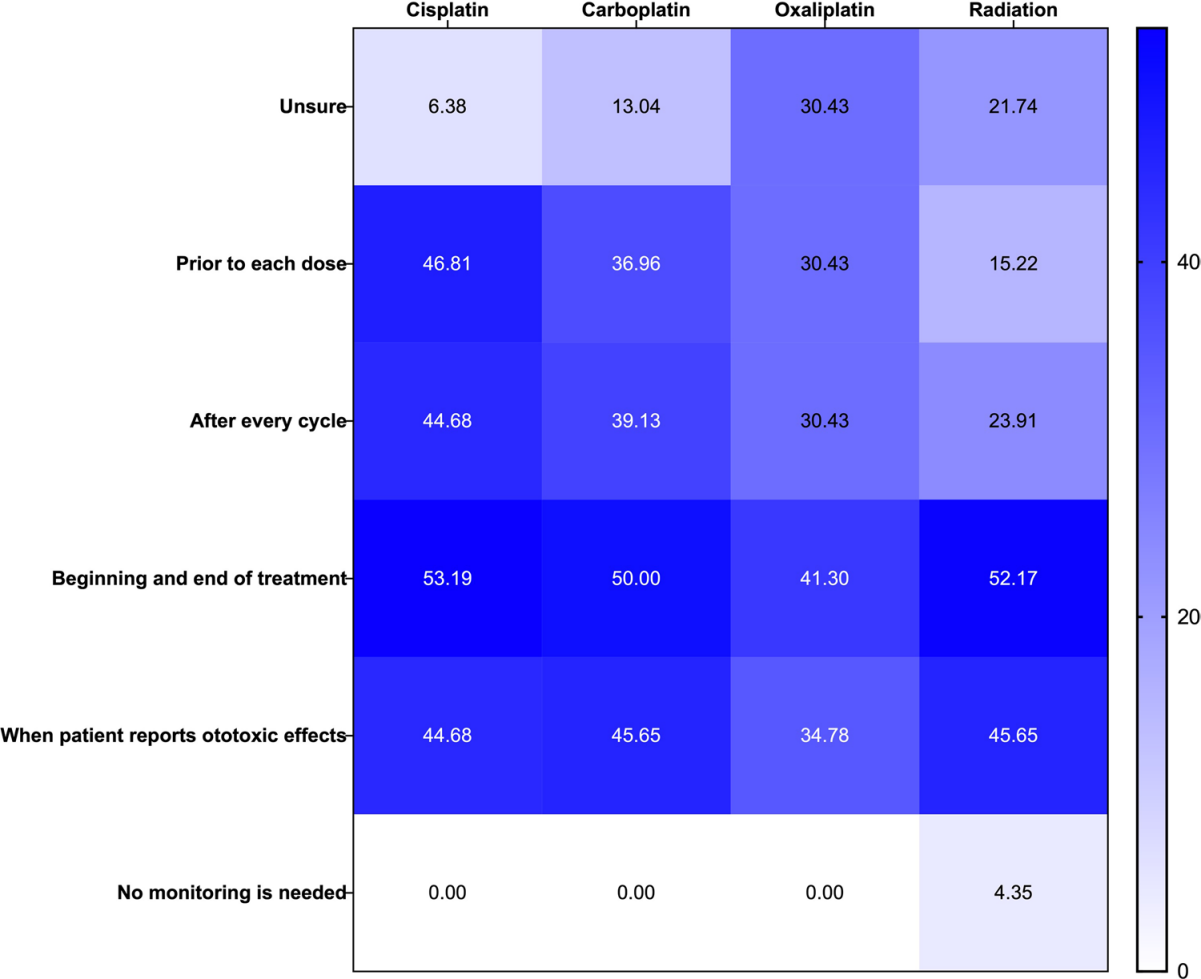
When to monitor for ototoxic hearing loss by ototoxic exposure type (columns). The numbers and color gradation in each panel indicate the percentage of respondents that selected a given symptom surveillance option (rows). *N = 47*

**Fig. 5:**
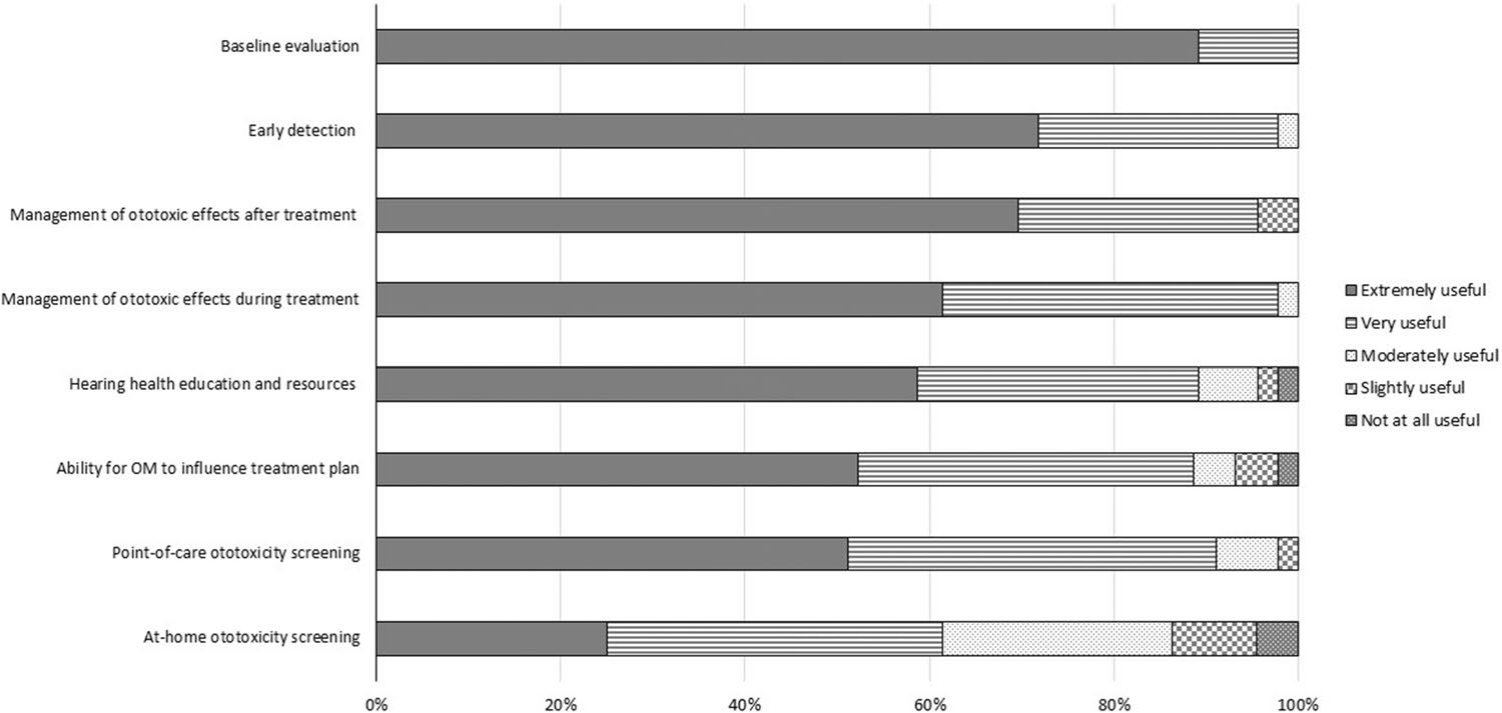
Baseline hearing evaluation, ototoxicity early detection, and management of ototoxic effects after treatment were considered the most useful aspects of ototoxicity management. This plot illustrates by shading type, the relative importance of various OtoM objectives (rows). *N = 46*

Respondents were asked to indicate the appropriate monitoring schedule for ototoxic medications based on drug type. Figure 4 shows that all respondents believed that some form of monitoring was needed for each ototoxic treatment except radiation. In addition, respondents were asked to rate the usefulness of different aspects of OtoM. Figure 5 displays stacked relative frequencies for each aspect of this question. Performing a baseline evaluation was rated as very useful to extremely useful by 100% of respondents, and approximately 97% of respondents rated the early detection of ototoxic effects as very useful to extremely useful. The only element that was rated as extremely useful by less than half of participants was at-home ototoxicity screening.

### Barriers associated with OtoM

When asked about the main barriers associated with OtoM implementation, respondents cited several including factors related to interdisciplinary communication (e.g., lack of collaboration between audiology and oncology), identifying patients (e.g., limited strategies to identify at-risk patients that should be included in their caseload), resources, and the absence of national protocols. Table 3 shows select quotations related to each of these themes.

**Table 3:** Key themes of reported barriers to OtoM practices. This table provides results of the qualitative analysis on the barriers to care identified by respondents (*N* = 63). The three themes that emerged from the analysis are provided in the left column. The middle column indicates the relevant CFIR construct (middle column) for each theme. The right column shows individual quotations that best illustrate the themes within a given CFIR domain

Theme	CFIR domain	Example quotations
Interdisciplinary communication and identifying patients	Inner setting	• Without an oncologist on site, it has been difficult to generate referrals or know which patients are receiving any of these ototoxic medications • Lack of communication between oncology and audiology • [Audiology] services not integrated as part of the treatment team with oncology
Resources	Inner setting	• Time and space to get patients seen before, after treatments, and after complaints of changes • Do not have ototoxic[ity] program specialist position • Perhaps if someone was on-call when ototoxic patients are identified
Lack of protocol	Outer setting	• A national standardized protocol would be helpful to encourage good communication between [audiology and oncology] departments • Scope of practice • No known protocol that both [audiology and oncology] departments follow

## Discussion

This survey study was conducted to characterize OtoM service gaps and barriers to care across the VA healthcare system from the audiologist’s perspective, with a focus on care provision for patients prescribed platinum-based chemotherapies and/or radiation for the treatment of cancer. The provision of OtoM is generally not standard practice for cancer patients despite the high rates of ototoxicity associated with platinum-based chemotherapy, the known adverse effects of ototoxicity on QoL, and the existence of position statements from medical professional organizations advocating OtoM [e.g., 26, 44]. The OtoMIC survey provided in Appendix A delivered useful information that, when combined with perspectives of VA oncology teams and patients, will facilitate our long-term goal to provide OtoM as a standard of care in the VA health care system.

The aggregated survey results indicate that audiology providers in the VA believe certain aspects of OtoM are “extremely useful” or “very useful” for patient’s receiving platinum-based drugs, and that some form of monitoring is necessary for this population. However, only about half of respondents reported having fully implemented these valued OtoM services for highly ototoxic, cisplatin, and carboplatin-containing chemotherapies. The most frequently reported barriers to implementing OtoM were a lack of integrated interdisciplinary care, referral issues, no standardized protocol to guide care, and insufficient resources. Lastly, there were inconsistencies in the respondents’ knowledge regarding ototoxicity symptoms, and differing opinions about the responsibilities of audiologists and oncologists for OtoM care. Audiologists were primarily deemed responsible for monitoring hearing during treatment and providing patients with hearing aids and rehabilitation, but the oncology team was considered the main source for informing patients about the risks of ototoxicity, monitoring patient-reported symptoms, counseling patients who develop ototoxicity, as well as referring patients for OtoM services.

The CFIR constructs used to guide each stage of this research were those that Damschroder et al. [36] determined through a meta-analysis to be most valuable for informing successful healthcare interventions. The CFIR outer setting indicates that implementing a clinical intervention requires an established patient need and prioritization among the clinical providers and organizational partners. This consensus is typically determined by rigorous evidence-based research; efficacy, and especially effectiveness data for the intervention program are particularly useful for influencing policies and budget decisions [45]. To date, such data are limited for OtoM [34, 44], highlighting a crucial need for future research in this area. The CFIR inner setting stipulates that the perspectives of healthcare providers are important to understand and consider when developing and implementing a clinical intervention because of the context these insights provide for why a protocol works or does not work. Additionally, provider perspectives can identify implementation characteristics of low or high value [37]. The findings of this study add to the available literature on healthcare providers’ perspectives on ototoxicity and its management. The CFIR characteristics of individuals revealed audiologists’ limited knowledge about the prevalence of ototoxicity, which highlights the need for additional education in this area.

The variation in the audiologists’ perspectives on when ototoxicity should be monitored may reflect the need to expand and refine existing guidance on OtoM protocols [26, 32]. Patient narratives [46] and data from a small clinical trial [34] indicate that patients value being able to access hearing healthcare during treatment with an ototoxic therapy and will follow-up to obtain new hearing aids or hearing aid adjustments. However, the clinical trial results revealed significant barriers to patients accessing OtoM in VA and suggest that a screening approach may help overcome some of these barriers [34]. In the general population, providing a hearing screen that demonstrates what sounds an individual cannot hear, is crucial for motivating a person with hearing loss to address their hearing healthcare needs [33, 47, 48]. The audiologists surveyed did not perceive hearing screening as a highly valued aspect of OtoM, indicating that probing to obtain further information about this perspective is needed to inform OtoM delivery methods. Additional outcome data in support of specific OtoM protocols and delivery methods will be crucial for refining and standardizing OtoM.

Survey results further indicate that barriers such as unclear referral pathways are exacerbated by a lack of confidence that oncology teams would value and utilize information about ototoxicity in their patients. Audiologists also point to the need for alignment across audiology and oncology care providers regarding their responsibilities for OtoM. While results indicated a small number of “unsure” responses, more respondents indicated that no provider was specifically assigned to key tasks or that multiple providers were. This underscores the need for interdisciplinary discussion, and perhaps formalized agreements, between care teams to clarify roles for different elements of OtoM. This could ensure that patients do not miss key opportunities to improve their long-term hearing, tinnitus, and balance outcomes.

Approximately 14 studies from five of the six global regions defined by WHO have collected patient data or surveyed health care providers on aspects of OtoM implementation in cancer patients. A list of these publications can be found in Appendix H with descriptions of methods and brief summaries of OtoM program prevalence and service gaps. Results of the present study are generally consistent with the results of these studies which collectively indicate that across the globe, ototoxicity is not consistently monitored or addressed, and that the burden is often placed on the patient to self-advocate for the recommended hearing healthcare. Barriers noted in these studies include a lack of communication and collaboration between audiology and oncology or primary care departments, oncology or primary care not prioritizing or referring patients for OtoM, lack of oncology provider knowledge of ototoxicity and its adverse effects, and a paucity of accepted protocols and trainings pertaining to OtoM. Themes related to facilitators also emerge from these studies including models of care that provide OtoM at the convenience of the patient (e.g., flexible scheduling of audiology appointments [44], hearing screening in the hospital infusion unit [34]).

Understanding how OtoM fits into the VA can inform integration into other healthcare systems and structures; however, applying similar analyses will be necessary to identify locally available resources and barriers to care in the many other contexts in which patients are treated with ototoxic drugs. The VA survey respondents may have been biased toward those who prioritize OtoM, in which case actual care gaps could be greater than indicated in this report. The current report also is limited to the VA audiology community. Ongoing work for this project includes surveying VA oncology teams (oncologists, oncology nurses, pharmacists, and patient coordinators). A better understanding of what purpose OtoM serves for the oncology team will be crucial for better integrating OtoM into oncology care. This report does not provide any data on the perspectives of patients receiving OtoM, a necessary element to ensure the care is relevant and valuable for the patient and to increase buy-in from a multidisciplinary team of providers as well as organizational leadership.

## Conclusion

The OtoMIC survey provides stakeholder perspectives to benchmark and address OtoM care gaps. The implications of this report add to the growing body of evidence that shows OtoM is not consistently provided for cancer patients across clinical settings, healthcare structures, and global regions. Perspectives from VA audiologists gained through this nationwide survey have clarified factors that are barriers to implementing OtoM in the largest organized healthcare system in the USA [41]. Collaboration between oncology and audiology is required to address shortcomings in current OtoM practices so that cancer survivors can have more control over their long term hearing health. OtoM can ideally provide education and counseling to bolster a patient’s healthcare literacy regarding ototoxicity, symptom surveillance for early detection of ototoxicity to inform cancer treatment decision making, rehabilitation to mitigate the effects of any unavoidable ototoxicity on quality of life, and strategies to address every-day communication difficulties.

### Supplementary Information

Below is the link to the electronic supplementary material.

Supplementary file1 (DOCX 439 KB)

## Footnotes

1 In this study, we administered the OtoMIC version of the survey tailored to audiologists and service chiefs, only.

## References

[1] Travis LB, Fossa SD, Sesso HD (2014). Chemotherapy-induced peripheral neurotoxicity and ototoxicity: new paradigms for translational genomics. J Natl Cancer Inst..

[2] Knight KRG, Kraemer DF, Neuwelt EA (2005). Ototoxicity in children receiving platinum chemotherapy: underestimating a commonly occurring toxicity that may influence academic and social development. J Clin Oncol.

[3] Rybak LP, Ramkumar V (2007). Ototoxicity. Kidney Int.

[4] Landier W, Knight K, Wong FL (2014). Ototoxicity in children with high-risk neuroblastoma: prevalence, risk factors, and concordance of grading scales—a report from the Children’s Oncology Group. JCO.

[5] Søgaard M, Thomsen RW, Bossen KS, Sørensen HT, Nørgaard M (2013). The impact of comorbidity on cancer survival: a review. Clin Epidemiol.

[6] Bielefeld EC, Tanaka C, di Chen G (2013). An Src-protein tyrosine kinase inhibitor to reduce cisplatin ototoxicity while preserving its antitumor effect. Anti-Cancer Drugs.

[7] Prayuenyong P, Taylor JA, Pearson SE (2018). Vestibulotoxicity associated with platinum-based chemotherapy in survivors of cancer: a scoping review. Front Oncol.

[8] Steyger PS (2021). Mechanisms of ototoxicity and otoprotection. Otolaryngol Clin North Am.

[9] Dillard LK, Lopez-Perez L, Martinez RX, Fullerton AM, Chadha S, McMahon CM (2022). Global burden of ototoxic hearing loss associated with platinum-based cancer treatment: a systematic review and meta-analysis. Cancer Epidemiol.

[10] Frisina RD, Wheeler HE, Fossa SD (2016). Comprehensive audiometric analysis of hearing impairment and tinnitus after cisplatin-based chemotherapy in survivors of adult-onset cancer. J Clin Oncol.

[11] Sheth S, Mukherjea D, Rybak LP, Ramkumar V (2017). Mechanisms of cisplatin-induced ototoxicity and otoprotection. Front Cell Neurosci.

[12] Dille MF, McMillan GP, Reavis KM, Jacobs P, Fausti SA, Konrad-Martin D (2010). Ototoxicity risk assessment combining distortion product otoacoustic emissions with a cisplatin dose model. J Acoust Soc Am.

[13] Cancer Statistics - NCI. Published April 2, 2015. Accessed September 12, 2022. https://www.cancer.gov/about-cancer/understanding/statistics.

[14] Pearson SE, Taylor J, Patel P, Baguley DM (2019). Cancer survivors treated with platinum-based chemotherapy affected by ototoxicity and the impact on quality of life: a narrative synthesis systematic review. Int J Audiol.

[15] Bezjak A, Lee CW, Ding K (2008). Quality-of-life outcomes for adjuvant chemotherapy in early-stage non-small-cell lung cancer: results from a randomized trial, JBR.10. J Clin Oncol.

[16] Ardeshirrouhanifard S, Fossa SD, Huddart R (2022). Ototoxicity after cisplatin-based chemotherapy: factors associated with discrepancies between patient-reported outcomes and audiometric assessments. Ear Hear.

[17] Falchook AD, Green R, Knowles ME (2016). Comparison of patient- and practitioner-reported toxic effects associated with chemoradiotherapy for head and neck cancer. JAMA Otolaryngol Head Neck Surg.

[18] Chou R, Dana T, Bougatsos C, Fleming C, Beil T (2011). Screening adults aged 50 years or older for hearing loss: a review of the evidence for the U.S. preventive services task force. Ann Intern Med.

[19] Lin FR, Yaffe K, Xia J (2013). Hearing loss and cognitive decline in older adults. JAMA Intern Med.

[20] Cunningham LL, Tucci DL (2017). Hearing loss in adults. N Engl J Med.

[21] Graydon K, Waterworth C, Miller H, Gunasekera H (2019). Global burden of hearing impairment and ear disease. J Laryngol Otol.

[22] Thomson RS, Auduong P, Miller AT, Gurgel RK (2017). Hearing loss as a risk factor for dementia: a systematic review. Laryngoscope Investig Otolaryngol.

[23] Cudmore V, Henn P, O’Tuathaigh CMP, Smith S (2017). Age-related hearing loss and communication breakdown in the clinical setting. JAMA Otolaryngol Head Neck Surg.

[24] Weinreich HM (2017). Hearing loss and patient-physician communication: the role of an otolaryngologist. JAMA Otolaryngol Head Neck Surg.

[25] Kros CJ, Steyger PS (2019). Aminoglycoside- and cisplatin-induced ototoxicity: mechanisms and otoprotective strategies. Cold Spring Harb Perspect Med.

[26] Konrad-Martin D, Poling GL, Garinis AC (2018). Applying U.S. national guidelines for ototoxicity monitoring in adult patients: perspectives on patient populations, service gaps, barriers and solutions. Int J Audiol.

[27] American Speech-Language-Hearing Association. Audiologic management of individuals receiving cochleotoxic drug therapy [Guidelines]. Available from www.asha.org/policy.

[28] American Academy of Audiology. Position statement and clinical practice guidelines: ototoxicity monitoring. Published online 2009. https://www.audiology.org/wp-content/uploads/2021/05/OtoMonGuidelines.pdf_539974c40999c1.58842217.pdf. Accessed 4 Jan 2023.

[29] American Academy of Otolaryngology-Head and Neck Surgery (AAO-HNS). Position statement: ototoxicity. Published 2015. https://www.entnet.org/resource/position-statement-ototoxicity/. Accessed 12 Sept 2022.

[30] World Health Organization. *World Report on Hearing*. World Health Organization; 2021. https://apps.who.int/iris/handle/10665/339913. Accessed 8 Sept 2021. 10.2471/BLT.21.285643 PMC8085630 33953438

[31] Professional Board for Speech, Language, and Hearing Professions. Audiological management of patients on treatment that includes ototoxic medications: guidelines. Published online 2018. https://www.hpcsa.co.za/Uploads/SLH/Guidelines%20for%20Audiological%20Management%20of%20Patients%20on%20Treatment%20that%20includes%20Ototoxic%20Medications.pdf.

[32] Meijer AJM, van den Heuvel-Eibrink MM, Brooks B (2021). Recommendations for age-appropriate testing, timing, and frequency of audiologic monitoring during childhood cancer treatment: an International Society of Paediatric Oncology Supportive Care Consensus Report. JAMA Oncol.

[33] Folmer RL, Saunders GH, Vachhani JJ (2021). Hearing health care utilization following automated hearing screening. J Am Acad Audiol.

[34] Konrad-Martin D, O’Connell Bennett K, Garinis A, McMillan GP (2021). A Randomized controlled trial using automated technology for improving ototoxicity monitoring in VA oncology patients. Am J Audiol.

[35] Santucci NM, Garber B, Ivory R, Kuhn MA, Stephen M, Aizenberg D (2021). Insight into the current practice of ototoxicity monitoring during cisplatin therapy. J Otolaryngol Head Neck Surg.

[36] Damschroder LJ, Aron DC, Keith RE, Kirsh SR, Alexander JA, Lowery JC (2009). Fostering implementation of health services research findings into practice: a consolidated framework for advancing implementation science. Implement Sci.

[37] Keith RE, Crosson JC, O’Malley AS, Cromp D, Taylor EF (2017). Using the Consolidated Framework for Implementation Research (CFIR) to produce actionable findings: a rapid-cycle evaluation approach to improving implementation. Implement Sci.

[38] Means AR, Kemp CG, Gwayi-Chore MC (2020). Evaluating and optimizing the consolidated framework for implementation research (CFIR) for use in low- and middle-income countries: a systematic review. Implement Sci.

[39] Kilbourne AM, Goodrich DE, Miake-Lye I, Braganza MZ, Bowersox NW. Quality Enhancement research initiative implementation roadmap: toward sustainability of evidence-based practices in a learning health system. Med Care. 2019;57 Suppl 10 Suppl 3:S286-S293. 10.1097/MLR.0000000000001144. 10.1097/MLR.0000000000001144 PMC6750196 31517801

[40] Atkins D, Kilbourne AM, Shulkin D (2017). Moving from discovery to system-wide change: the role of research in a learning health care system: experience from three decades of health systems research in the veterans health administration. Annu Rev Public Health.

[41] Department of Veterans Affairs (VA), Maintaining Internal Systems and Strengthening Integrated Outside Networks, (MISSION) Act Section 505(b). Annual Report. Published online 2020. Accessed September 9, 2022. https://www.va.gov/EMPLOYEE/docs/Section-505-Annual-Report-2020.pdf.

[42] Veterans Benefits Administration. Annual benefits report fiscal year 2021. Published online 2021. Accessed July 29, 2022. https://www.benefits.va.gov/REPORTS/abr/docs/2021-abr.pdf.

[43] Braun V, Clarke V (2006). Using thematic analysis in psychology. Qual Res Psychol.

[44] Paken J, Govender CD, Pillay M, Sewram V (2022). Cisplatin-associated ototoxicity: perspectives from a single institution cervical cancer cohort and implications for developing a locally responsive monitoring programme in a public healthcare setting. BMC Health Serv Res.

[45] Durlak JA, DuPre EP (2008). Implementation matters: a review of research on the influence of implementation on program outcomes and the factors affecting implementation. Am J Community Psychol.

[46] Clark KD, Garinis AC, Konrad-Martin D (2021). Incorporating patient narratives to enhance audiological care and clinical research outcomes. Am J Audiol.

[47] Yueh B, Collins MP, Souza PE (2010). Long-term effectiveness of screening for hearing loss: the screening for auditory impairment–which hearing assessment test (SAI-WHAT) randomized trial. J Am Geriatr Soc.

[48] Donahue A, Dubno JR, Beck L (2010). Guest editorial: accessible and affordable hearing health care for adults with mild to moderate hearing loss. Ear Hear.

