# Supplementary material for: Audiologists’ perceived value of ototoxicity management and barriers to implementation for at-risk cancer patients in VA: the OtoMIC survey
Source: J Cancer Surviv. 2023 Feb 2;17(1):69–81. doi: 10.1007/s11764-022-01316-7 (PMC9892665; doi:10.1007/s11764-022-01316-7)
Supplement: Supplementary file 1 — Supplementary file1 (DOCX 439 KB) [file 11764_2022_1316_MOESM1_ESM.docx]

**Appendix A.** Individual questions from the “*Ototoxicity Management Through Interdisciplinary Care (OtoMIC)*” survey for audiology and oncology clinicians and their leadership. Questions are shown mapped to project objectives, the Consolidated Framework for Implementation Research (CFIR) constructs, and the assessment approach. The audiology professionals surveyed for this report were presented the subset of questions as indicated on the survey. Minor changes to improve the survey questions occurred following its administration and the final version is shown below*.*

**Objectives of the OtoM-IC survey:** The objective of the OtoMIC survey is to solicit perspectives on the following topics: a) clinic, provider, and patient characteristics (e.g., years of practice, chief auditory complaint and age of the patient population); b) numbers of patients prescribed ototoxic medications at the respondent’s care setting; c) provider awareness of the prevalence of ototoxicity; d) provider awareness of symptoms of ototoxicity; e) provider awareness of patient risk factors for ototoxicity; f) the magnitude of the provider’s patient caseload with auditory or vestibular changes due to ototoxicity; g) who is responsible for specific elements of OtoM in the respondent’s care setting; h) the value of specific elements of an OtoM program; i) current ototoxicity care processes/prioritization of OtoM; j) how patients access OtoM services; k) perspectives on audiologists’ scope of practice; l) if the respondent’s institution follows an OtoM protocol; and m) if the respondent’s practice setting is open to updating OtoM processes and standard practices.

| Ototoxicity Management Through Interdisciplinary Care (OtoMIC) Survey | | | | | |
| --- | --- | --- | --- | --- | --- |
| **CFIR Construct** | **Question** | **Objective** | | **Question Type** | |
| The **purpose of this survey** is to determine how best to provide hearing healthcare to patients receiving ototoxic drug therapies in the VA. Questions will ask about your experience with, knowledge of, and priorities regarding ototoxicity risk factors, symptoms, and management protocols.  **Instructions:** Please answer each question by selecting the best answer. If you are unsure about how to answer a question, please give the best answer you can. | | | | | |
| **Inner setting** - Demographics  (Structural characteristics) | 1. *What is your primary medical specialty?*   - Audiology Chief - Audiologist - Other (text box) | | a | | Multiple choice |
| **Demographics** | 2. *What is your terminal degree?*   - AuD - PhD - MS or MA - Other (text box) | | a | | Multiple choice |
| **Demographics** | 3. *What is your VA practice setting?*   - VA Medical Center - Community Based Outpatient Clinic (CBOC) - Both | | a | | Multiple choice |
| **Demographics** | 4. *What is your practice location? (Please enter your VISN and 3 letter city abbreviation.)* | | a | | Open ended qualitative |
| **Demographics** | 5. *How many years have you been practicing in your field?*   - Less than a year - 1 to 3 years - 4 to 6 years - 7 to 10 years - 11 to 20 years - More than 20 years | | a | | Multiple choice |
| This section asks details about your patient population. If you are unsure of an answer, please choose the best/closest answer. | | | | | |
| **Outer setting** – Patient needs and resources  (Prevalence) | 6. *Approximately how many patients per month…*   - …do you provide audiological services for who are receiving _____? (Audiologist) - …does your service provide audiological services for who are receiving _____? (Audiology Chief) - …do you treat with _____? (Oncologist)   6. *If _____ is part of the treatment, do oncology patients typically receive audiological monitoring for ototoxicity?* (Nurse; Oncology Chief; Oncologist)  Rows   - Cisplatin - Carboplatin - Oxaliplatin - Radiation   Columns   - Less than 5 - 5 to 10 - 11 to 15 - 16 to 20 - More than 20 - Unsure | | b | | Multiple selection (matrix) |
| **Outer setting** – Patient needs and resources  (Prevalence) | 7. *Approximately what percentage of your patients have had the following as a result of an ototoxic agent?*   - New or increased hearing loss - New or increased tinnitus - New or increased balance problems - Decreased quality of life - Unsure | | f | | Sliding scale percentage or select |
| **Individuals involved** – Evaluation of knowledge  (Prevalence) | 8. *What is the prevalence of ototoxic hearing loss among oncology patients receiving the following ototoxic agents?*   - Cisplatin - Carboplatin - Oxaliplatin - Radiation - A combination of ototoxic agents - Unsure | | c | | Sliding scale percentage or select |
| This section of questions asks about your knowledge as a care provider about ototoxicity, including symptoms, risk factors, and monitoring practices. Please answer each question to the best of your ability. | | | | | |
| **Individuals involved** – Evaluation of knowledge | 10. *Which of the following are known risk factors for ototoxicity?* ***(Select all that apply.)***   - Young age (Less than 5 years) - Older age - Inflammation - Hydration status - Dose and duration of ototoxic therapy - Renal insufficiency or insult - Hepatic failure - Metastasis - Good pre-treatment hearing - Poor pre-treatment hearing - Exposure to head and neck radiation - Concomitant intake of other ototoxic agents - Family history of ototoxicity - Poor diet/nutrition - Unsure - Other (text box) | | e | | Multiple selection |
| **Individuals involved** – Evaluation of knowledge | (for Oncologist, Nurse, and Oncology Chief)  11. *For the following scenario please select the answer(s) that best reflect your action plan.*   - A family member of a patient brings up that the patient has had a hard time following conversations in a noisy environment since their last cycle of cisplatin. - Refer to audiology for hearing, tinnitus or balance management - Consider changing the dosage of cisplatin in the next cycle - Consider changing the agent in the next cycle - Provide counseling - Increase the frequency of ototoxicity monitoring - No treatment change   Other (text box) | | i, k | | Multiple selection |
| **Individuals involved** – Evaluation of knowledge | (for Oncologist, Nurse, and Oncology Chief)  12. *For the following scenario please select the answer(s) that best reflect your action plan.*   - A patient reports ringing in their ears before they are supposed to start a new cycle of carboplatin and radiation. - Refer to audiology for hearing, tinnitus or balance management - Consider changing the dosage of carboplatin in the next cycle - Consider changing the agent in the next cycle - Provide counseling - Increase the frequency of ototoxicity monitoring - No treatment change   Other (text box) | | i, k | | Multiple selection |
| **Individuals involved** – Evaluation of knowledge | (for Oncologist, Nurse, and Oncology Chief)  13. *For the following scenario please select the answer(s) that best reflect your action plan.*   - The audiologist has confirmed that after receiving a cumulative dose of 170 mg/m2 of cisplatin a patient has had a significant hearing shift compared with their pre-treatment baseline evaluation. This patient will require a hearing aid. The patient is concerned about the persistent ringing and loss of hearing they have experienced since their last dose of cisplatin and is worried about progression of the hearing loss with further treatment. The tumor response to the treatment to the drug has been good. - Refer to audiology for hearing, tinnitus or balance management - Consider changing the dosage of cisplatin in the next cycle - Consider changing the agent in the next cycle - Provide counseling - Increase the frequency of ototoxicity monitoring - No treatment change   Other (text box) | | i, k | | Multiple  selection |
| **Individuals involved** – Evaluation of knowledge | 14. *What do you believe is an appropriate schedule of ototoxicity monitoring tests for the results to be useful/actionable for you and your patients?* ***(Select all that apply.)***  *For clarification: "Treatment" refers to entire regimen; "cycle" refers to the repeated portion of the regimen.*  Rows   - Cisplatin - Carboplatin - Oxaliplatin - Radiation   Columns   - No monitoring is needed if patient is counseled about risks - Beginning and end of treatment - Prior to administration of each dose - After every cycle - When patient reports ototoxic effects/symptoms - Unsure | | i | | Multiple selection (matrix) |
| **Individuals involved** – Evaluation of knowledge | 15. *Do you have a preferred ototoxicity monitoring schedule not reflected in the previous question?* | | i | | Open ended qualitative |
| **Individuals involved** – Evaluation of knowledge | 16. *Please rank the importance of the following factors for determining the appropriate schedule of ototoxicity monitoring tests (with 1 as the most important).*  Rows   - Cost - Feasibility (availability of patients, staff, and equipment) - Patient preference - Patient’s current health status - Relevance of test results to treatment plan - Relevance of test results to rehabilitation plan   Columns   - 1 - 2 - 3 - 4 - 5 - 6 | | i | | Ranking (1-6) |
| This section addresses current practices related to ototoxic symptoms including hearing loss, tinnitus, and vestibular effects. | | | | | |
| **Inner setting** - Network and communications  (Current methods/practice) | 17. *At your facility, which clinical team member is responsible for the following?* ***(Select all that apply.)***  Rows   - Inform patients of the risks for ototoxicity - Monitor patient reported symptoms - Monitor hearing during treatments with ototoxic agents - Counselling patients who develop hearing loss as a result of ototoxic agents - Providing patients with hearing aids and rehabilitation   Columns   - Audiologist - Oncology team - No specifically assigned provider - Unsure | | g | | Multiple selection (matrix) |
| **Inner setting** – Network and communications  (Current methods/practice) | 18. *For patients receiving each of the following agents…*   - *…do you routinely:* (Audiologist) - …*does your service routinely:* (Audiology Chief)   Rows   - Perform baseline evaluation prior to starting oncology treatment? - Monitor for hearing changes? - Screen for tinnitus? - Screen for vestibular changes? - Perform a follow-up examination after the conclusion of oncology treatment?   Columns   - Cisplatin   - Yes   - Sometimes   - No - Carboplatin   - Yes   - Sometimes   - No - Oxaliplatin   - Yes   - Sometimes   - No - Radiation   - Yes   - Sometimes   - No | | g, i | | Multiple choice (matrix) |
| **Inner setting** –  Network and communications  (Current methods/practice) | 19. *How do patients at your site access ototoxicity management?* ***(Select all that apply.)***   - Referral from Pharmacy - Referral from Primary Care - Referral from Audiologist - Referral from Oncology Team - Self-referral - Unsure - Other (text box) | | j | | Multiple selection |
| **Inner setting** - Network and communications  (Evaluation of knowledge for oncologists) | 20. *Which of the following interventions falls under the audiologist’s scope of practice?* ***(Select all that apply.)***   - Modification of medication - Prescribing hearing aids and other assistive devices - Balance management - Tinnitus management - Education and counseling about risks and impacts of ototoxicity - Pre- and post-treatment hearing tests - Unsure - Other (text box) | | k  (g, i) | | Multiple selection |
| The last set of questions is intended to gather information on the climate for change regarding new or updated ototoxicity management protocols.  *For clarification: Ototoxicity monitoring refers to symptom surveillance; ototoxicity management refers to symptom surveillance, rehabilitation and/or treatment.* | | | | | |
| **Inner setting** - Implementation climate  (Capacity for change) | 21. *Please rank the importance of managing the following treatment side effects.****(Click and drag each choice up or down to change the ranking where 1 is the most important.)***   - Hearing loss - Loss of taste - Nausea - Neuropathy - Sexual dysfunction - Sleep disturbance - Tinnitus - Vestibular (balance) dysfunction - Other (text box) | | i, h | | Ranking (1-8/9) |
| **Inner setting** – Implementation climate | 22. *What barriers exist to implementing ototoxicity monitoring/management at your facility?* | | h, m | | Open ended qualitative |
| **Inner setting** - Implementation climate | 23. *What changes and/or additional resources would increase or improve access to ototoxicity management at your facility?* | | h, m | | Open ended qualitative |
| **Inner setting** - Implementation climate | 24. *Which of those changes would receive strong leadership support and or clinician support at your facility?* | | h, m | | Open ended qualitative |
| **Inner setting** - Implementation climate | 25. *If an ototoxicity management program is available at your site, to what extent do you (would you) value the following?*  Rows   - Early detection of hearing loss, tinnitus, and balance problems - Ability for ototoxicity monitoring to influence the cancer treatment plan - Hearing health education and counseling resources to the patient/family - Management of hearing loss, tinnitus and/or balance issues **during** treatment with ototoxic agent - Management of hearing loss, tinnitus, and/or balance issues **after** treatment with ototoxic agent - Baseline evaluation prior to starting oncology treatment - Point-of-care (e.g., in oncology infusion unit) ototoxicity screening - Point-of-care (e.g., in oncology infusion unit) and/or same-day oncology consult - At-home ototoxicity screening - Prediction of ototoxic risk (change in hearing) prior to treatment with ototoxic agent   Columns   - Extremely useful - Very useful - Moderately useful - Slightly useful - Not at all useful | | h | | Multiple choice (matrix) |
| **Inner setting –** Implementation climate | 26. *Do you have any additional comments or recommendations related to ototoxicity management at your facility?* | | h | | Open ended qualitative |
| This survey is licensed but is freely available for most uses. If you would like to use this survey or any portions of it, please contact Dawn Konrad-Martin (dawn.martin@va.gov). | | | | | |

**Appendix B.** **A respondent from each VA region participated in the OtoMIC survey.** This figure shows a map of the United States with VA integrated service network regions (VISN) outlined and numbered. Stars indicate the service location of each respondent (total responded, N=63; completed survey, N=61; responded with location, N=57; responded with general qualitative input only, N=2).


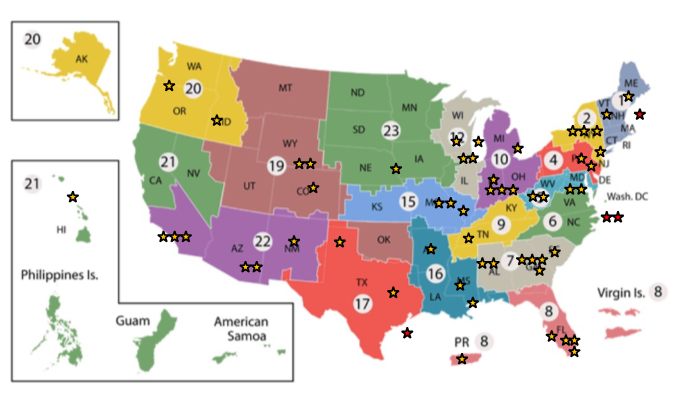


**Appendix C. For each category of ototoxic cancer treatment, most respondents/clinics reported that they saw fewer than 5 patients per month.** The reported number of patients seen by audiology each month for OtoM is shown by treatment type (x axis) as the relative frequency across responses to this survey question. (N=54).

**Appendix D. There is a wide distribution of the reported prevalence of ototoxicity symptoms across the respondents’ caseloads**. Respondents believed that receiving an ototoxic agent was responsible for decreased quality of life in up to 80% of their patients and that symptoms of increased hearing loss, tinnitus and balance problems were on average experienced in 50-65% of their patients on an ototoxic treatment. When compared with the literature, balance problems from any treatment were reported at a higher frequency while symptoms associated with cisplatin exposure were reported at a lower frequency than expected.

| Approximately what percentage of your patients have had the following as a result of an ototoxic agent (N = 28)? | |
| --- | --- |
| New or increased hearing loss | 17.6% (0%-50%) |
| New or increased tinnitus | 20% (0%-65%) |
| New or increased balance problems | 16.8% (5%-50%) |
| Decreased quality of life | 29.3% (0%-80%) |
| What is the prevalence of ototoxic hearing loss among oncology patients receiving the following ototoxic agents (N=20)? | |
| Cisplatin | 31.8% (5%-100%) |
| Carboplatin | 15.8% (5%-50%) |
| Oxaliplatin | 5% (5%-5%) |
| Radiation | 16% (5%-50%) |

**Appendix E. Perspectives on audiology scope of practice.** Respondents could select more than one answer. Results are given in terms of the percentage of respondents that indicated an item was in their scope (N=45). Not all audiologists felt it was within their scope of practice to provide education about ototoxicity.


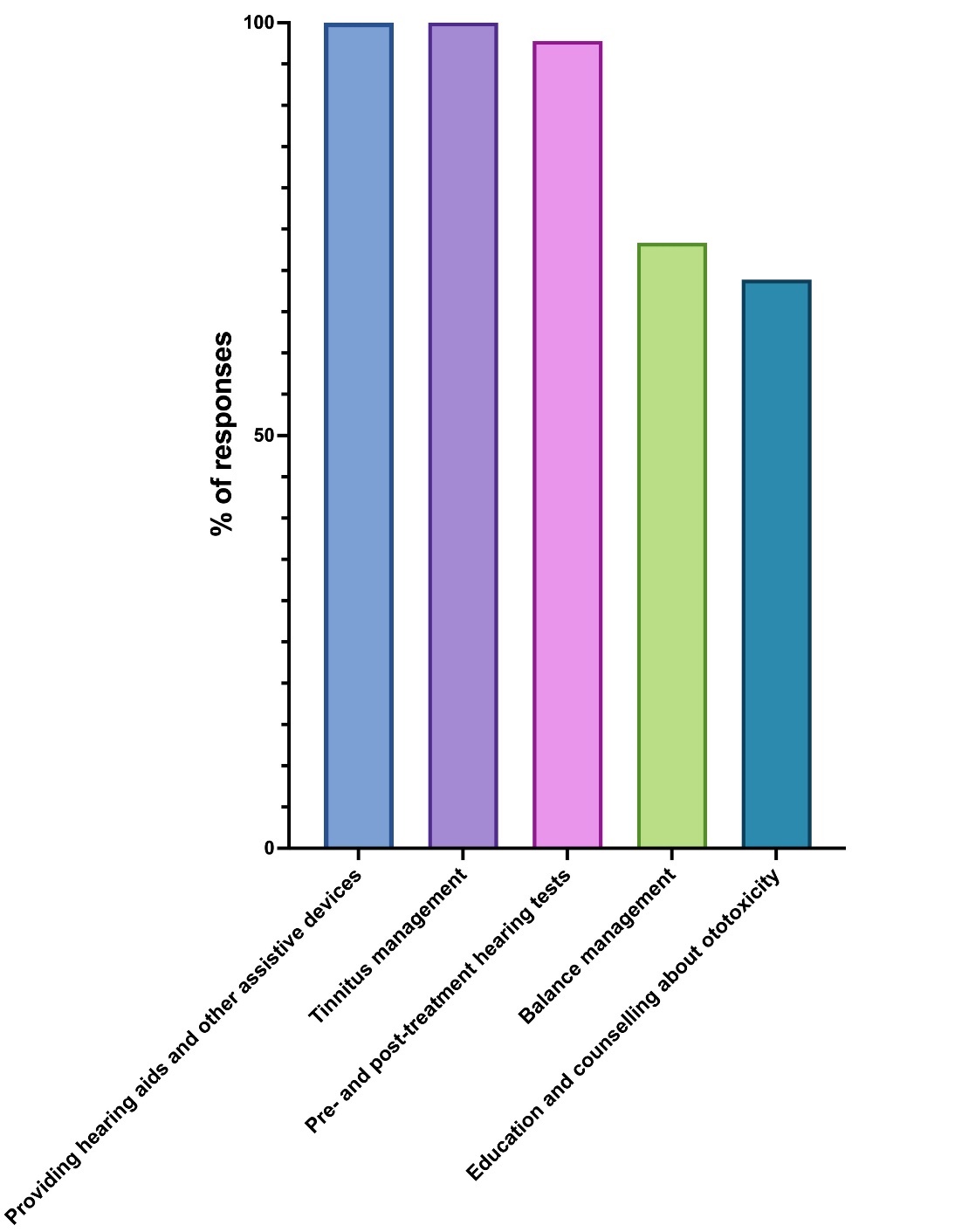


**Appendix F.** **Ranking of non-lethal side effects of treatment with chemotherapeutic agents according to the importance of managing each symptom**. A ranking of 1^st^ was the most important and 8^th^ was the least important. Hearing loss and vestibular dysfunction were ranked as the most and second most important side effects to manage, respectively. N=46.

**Appendix G. Referral sources for ototoxicity management.** Results of Question 19 indicate that the oncology service provides nearly 90% of referrals for ototoxicity management of VA cancer patients.

| How do patients at your facility access ototoxicity monitoring and/or management (N=46)? | |  |  |
| --- | --- | --- | --- |
|  |  |  |  |
| Referral from oncology team | 41 (89%) |  |  |
| Referral from primary care | 15 (33%) |  |  |
| Referral from audiology | 11 (24%) |  |  |
| Self-referral | 11 (24%) |  |  |
| Other | 4 (9%) |  |  |
| Unsure | 4 (9%) |  |  |
| Referral from pharmacy | 3 (7%) |  |  |

**Appendix H: List of key references on the current state of ototoxicity management (OtoM) practices across the world.** An asterisk (*) indicates that the publication includes provider surveys and interviews. A plus symbol (+) indicates that the study contains prospective and/or retrospective evaluations of efficacy.

| **Author** | **Year** | **Country**  **(Region)** | **Methods** | **Prevalence or Knowledge of Ototoxicity Management** | **Ototoxicity Management Service Gaps** |
| --- | --- | --- | --- | --- | --- |
| de Andrade, Khoza-Shangase, Hajat [51] | 2009 | **South Africa (African Region)*** | Surveyed 10 oncologists from 2 public hospitals in Gauteng. | No respondents reported having an OtoM protocol and only 50% reported referring patients for OtoM. All oncologists reported that chemotherapy can impact hearing, but 20% were unsure of what the symptoms were. Oncologists reported varied understanding of the physical effects of chemotherapy on the ear. 80% of oncologists reported that they provide patient education on ototoxicity, but do not collect relevant case history information on risk factors. | -Lack of provider knowledge  -No audiologists as a part of the oncology team  -Lack of OtoM protocols |
| Ehlert, Heinze, Swanepoel [52] | 2022 | **South Africa (African Region)*** | Surveyed public and private providers (including general practitioners, oncologists, nurses, pharmacists, and audiologists) and audiologists at outpatient referral clinics | No oncology providers reported knowledge of OtoM protocols. All oncology providers and 14% of audiologists stated that there was a lack of OtoM best practice guidelines. 14% of audiologists reported knowledge of best practice guidelines, but 43% of audiologists stated they follow best practice guidelines. Audiologists and oncology providers agreed that the purposes of OtoM include early identification, education, and timely intervention, but disagreed about terminating or adjusting treatment. | -Lack of effective OtoM referral system  -Need for a multidisciplinary approach and a simplified national protocol  -Environmental noise interferes with testing outside of ideal test settings |
| Garinis, Cornell, Allada, Fennelly, Maggiore, Konrad-Martin [53] | 2018 | **USA (Region of the Americas)*** | Physicians (N=3 pulmonologists and N=2 medical oncologist) surveyed about their perspectives on the provision of OtoM | The physicians had widely different approaches to ototoxicity monitoring, from audiology referrals to patient self-report. All physicians indicated that ototoxicity is a  potential problem for their patients; that monitoring hearing is  important for their caseload; and  that modification of a treatment regimen would be considered if  alternative treatment options were available. | Stakeholder perspectives (physician input) on ototoxicity monitoring must be considered to optimize inter-disciplinary care |
| Khoza-Shangase & Jina [48] | 2013 | **South Africa (African Region)*** | Surveyed general practitioners (N=28) to determine their perceptions regarding OtoM | General practitioners have easy access to audiological services, but do not all utilize these services | -General practitioners do not treat ototoxicity as a medical priority    -There is a lack of communication between Audiologists and general practitioners |
| Khoza-Shangase & Masondo [54] | 2020 | **South Africa (African Region)*** | Surveyed 21 audiologists about current OtoM | >2/3 engage in some form of OtoM but implementation of OtoM does not align with (inter)national guidelines and standards | Translating OtoM knowledge and guidelines into practice is lacking |
| Konrad Martin, Poling, Garinis, Ortiz, Hopper, O’Connell Bennett, Dille [26] | 2018 | **USA (Region of the Americas)*** | Interviewed and surveyed OtoM programs (N=5) about their characteristics, barriers, and facilitators | N/A all individuals surveyed were knowledgeable about OtoM | -The 5 programs reported similar concerns and perceived barriers related to the logistics of accessing and testing patients  -Successful programs were well-integrated with the services that provided care with ototoxic drugs (oncology, infectious disease) |
| Konrad-Martin, O’Connell Bennett, Garinis, McMillan [35] | 2021 | **USA (Region of the Americas)^+^** | A randomized controlled trial to determine the efficacy of automated ototoxicity monitoring compared with usual care in Veterans receiving cisplatin chemotherapy | 46% and 76% of the Veterans incurred an ototoxic hearing loss 35 days and 365 days post-randomization, respectively. Most patients had substantial hearing loss and self-perceived hearing handicap even before treatment with an ototoxic drug. | -Poor adherence (4.5%) to ototoxic monitoring through a typical service delivery approach  -Providing OtoM in the infusion unit increased adherence to recommended ototoxicity monitoring through improved access  -Patients with identified hearing loss had a high rate of uptake of recommended audiology services  -One patient in each arm had treatment changed due to ototoxicity |
| Kuchya, Tode, Sachdeva, Salankar [55] | 2019 | **India (South-East Asian Region)*** | Surveyed medical providers (N=86) about their knowledge and understanding of ototoxicity | Most respondents were aware of and had good knowledge of ototoxic drugs. Respondents saw an average of 5-6 new ototoxicity cases each week. | Not Addressed |
| Maru & Malky [56] | 2018 | **UK (European Region)*** | Survey of audiologists and general practitioners (N=134) familiar with ototoxicity to assess current OtoM practices and patient pathways | 72% reported the absence of OtoM protocols within their center/ | -Lack of a multidisciplinary team    -General practitioners do not actively and regularly engage in OtoM |
| Paken, Govender, Pillay, Sewram [49] | 2020 | **South Africa (African Region)*** | Surveyed 7 oncologists, 9 nurses, and 13 pharmacists and interviewed 4 audiologists, all from KwaZuluNatal hospital | 82% of sample considered audiology to be part of the oncology team, but audiologists reported that oncology does not refer for ototoxicity. Oncologists and nurses reported that reduced hearing sensitivity and ear pain were common symptoms following cancer treatment, but there was no ototoxicity protocol or OtoM program in place. All respondents reported varied and incomplete knowledge of OtoM practices and ototoxicity risk. | -OtoM is not included as a part of oncologic care  -Lack of OtoM protocols and program  -Lack of awareness of OtoM roles and responsibilities by provider groups  -Incomplete provider knowledge of ototoxicity and OtoM best practices |
| Paken, Govender, Pillay, Sewram [44] | 2022 | **South Africa (African Region)*^+^** | Surveyed 80 patients with cervical cancer, 6 clinicians, 8 oncology nurses, 9 pharmacists, and 9 radiotherapists in KwaZuluNatal hospital. Also analyzed hospital records | 91% of healthcare providers and 89% of patients found OtoM beneficial. Clinicians were the main source of patient education on ototoxicity and referrals. | OtoM is time-consuming for patients and providers, who already have tight schedules    -Patient guidance on ototoxicity is lacking  -Cost of setting up and staffing OtoM clinics is high |
| Santucci, Garber, Ivory, Kuhn, Stephen, Aizenberg [36] | 2021 | **USA (Region of the Americas)^+^** | Analyzed patient records (N=379) at UC Davis Medical Center between 2014 -2017 | 51% of patients were counseled regarding ototoxic risks; 24% of patients had documented hearing complaints. Only 4.5% of patients received an audiogram during cisplatin treatment. | -Poor adherence to national OtoM guidelines at a large academic medical center  -Lack of education and collaboration between otolaryngology, audiology, and medical oncology |
| Steffens, Venter, O’Beirne, Kelly-Campbell, Gibbs, Bird [57] | 2014 | **New Zealand (Western Pacific Region)*** | Interviewed (N=16) audiologists and (N=16) oncologists (N=7) regarding ototoxicity management and implementation of a national guideline | There was a comprehensive understanding of ototoxicity across both disciplines but limited familiarity with ototoxicity management protocols. | Lack of national guidelines and training |
| Wium & Gerber [58] | 2016 | **South Africa (African Region)^+^** | Surveyed 90 physicians from hospital departments that are likely to encounter ototoxic hearing losses (neonatal intensive care units, ENT, internal medicine) | 53% of participants could identify 80% or more of ototoxic medications from a provided list. 89% indicated that they needed more information about otoprotective strategies. Only 16% of providers routinely provide patient education and 18% routinely refer to audiology. | -Insufficient time and provider knowledge of ototoxicity  -Few referrals for OtoM. |
